# Malignant extra-adrenal pancreatic paraganglioma: case report and literature review

**DOI:** 10.1186/1471-2407-13-486

**Published:** 2013-10-20

**Authors:** Bilal O Al-Jiffry, Yasir AlNemary, Samah H Khayat, Moutaz Haiba, Mohammed Hatem

**Affiliations:** 1 Department of Surgery, College of Medicine and Medical Sciences, Taif University, PO Box 888, Taif 21947, Kingdom of Saudi Arabia; 2 Department of Surgery, Al-Hada Military Hospital, PO Box 1347, Taif, Kingdom of Saudi Arabia; 3 Department of Pathology, Al-Hada Military Hospital, PO Box 1347, Taif, Kingdom of Saudi Arabia

**Keywords:** Pancreatic tumors, Extra adrenal paraganglioma, Malignant paraganglioma, Functional paragangiloma, Pancreatic paragangilioma

## Abstract

**Background:**

Pancreatic paragangliomas are rare tumors, with only 16 reported cases to date. One of these cases demonstrates metastasis to lymph node, while another case was functional, however, none of these cases showed malignant and large, pancreatic paraganglioma with marked invasion. Also another unique feature was the age of our patient compared to the average reported ages in published literature (42–85 years).

**Case presentation:**

A 19-year-old woman presented with a one-year history of intermittent abdominal pain. Physical examination showed a palpable mass in the right upper abdomen, but initial laboratory results were within normal ranges; tumor markers (CEA, AFP, and CA19-9) were negative. An abdominal and pelvic computed tomography (CT) scan showed a well-defined retroperitoneal para-aortic mass. The CT scan revealed that the surrounding lymph nodes were not enlarged, but the liver showed evidence of parenchymal infiltration. Intraoperatively, a large, firm tumor originating from the head of pancreas was found pushing on the caudate hepatic lobe and the inferior vena cava (IVC). The tumor was resected through a pancreaticoduodenectomy, involving segment VI of the liver and a small segment of the IVC. The blood pressure spiked (>220 mm Hg) when the tumor was manipulated during the operation. The final pathology report showed a 9-cm tumor with lymphovascular invasions; immunohistochemistry was positive for synaptophysin and chromogranin. All resection margins were negative and 1/15 lymph nodes was positive for metastasis. Post-operative recovery was unremarkable. One month after discharge, the patient was re-admitted with abdominal pain and found to have an abdominal collection at the resection site, which was drained under CT guidance. She received a therapeutic dose of I^131^-metaiodobenzylguanidine (MIBG). Follow-ups showed the absence of recurrence, and she has remained disease free.

**Conclusion:**

This patient was an extraordinary example of a rare tumor. Even more remarkable was that the tumor was malignant with lymph node invasion. To our knowledge, a case similar to that presented here has not been previously reported in the literature.

## Background

Paragangliomas are rare tumors of neural crest origin, with a malignancy rate of approximately 10% and a 5-year survival rate of <50%. However, in the absence of metastasis, the tumors are considered benign [1,2]. The average age reported in the literature for the detection of extra-adrenal pancreatic paragangliomas is 67 years (range, 42–85 years) [1]. Primary paragangliomas of the pancreas are extremely rare, with only 16 cases having been reported in the literature [3,4]. In the previously reported 16 primary pancreatic paragangliomas, there was only one case that was considered malignant with lymph node metastasis and one case that showed hormonal activity [1,5]. In this report, we present a malignant pancreatic paraganglioma in a 19-year-old woman.

## Case presentation

**Figure 1:**
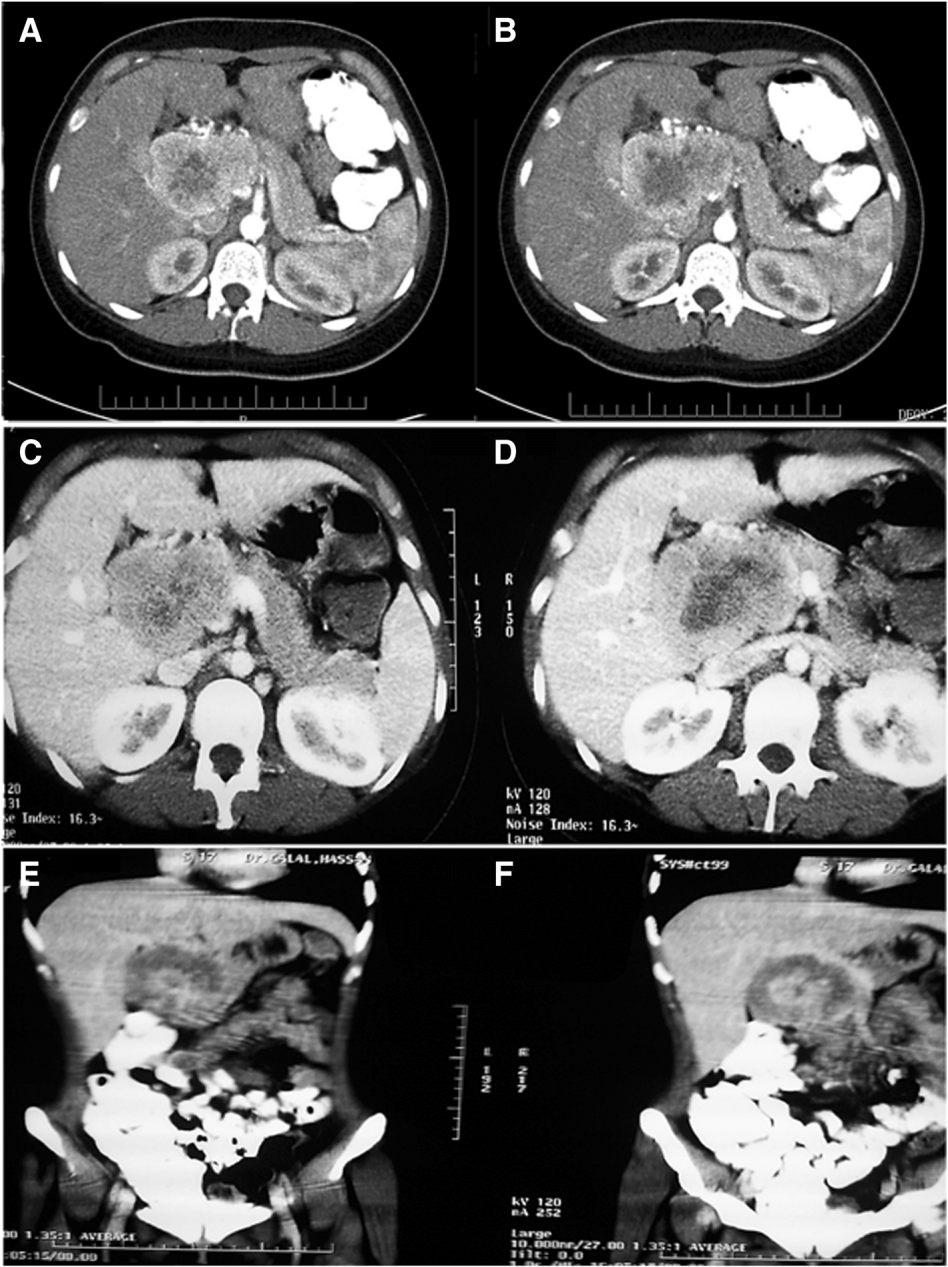
**A computed tomography scan demonstrating a large, well defined pancreatic mass. A-D** shows the mass in the pancreatic head and ancinate process with marked enhancement in the arterial phase. **E-F**: The lesion was anterior to the aorta and inferior vena cava, anteromedial to the right kidney, abutting the fundus of the gall bladder, and reaching the level of porta hepatis.

A 19-year-old woman presented in March 2010 with vague, recurrent upper abdominal pain. The pain had been occurring for approximately one year and had increased in frequency and severity. A physical examination revealed the presence of a large, palpable firm mass, which was slightly mobile, with an irregular outline, in the right upper abdomen. There were no other remarkable findings. An initial laboratory investigation returned results that were within normal ranges, while the tumor markers CEA, AFP, and CA19-9 were not elevated. An abdomino-pelvic computed tomography (CT) scan (Figure 1) showed a well-defined kidney-shaped retroperitoneal para-aortic mass. The mass measured 9 cm × 5 cm × 9.5 cm and showed peripheral enhancement after injection of an intravenous contrast agent with a relatively central hypodense pattern and abundant vascularity. The lesion originated from the posterior aspect of the head and neck of the pancreas. Figures 1A and B show the mass in the pancreatic head and ancinate process with marked enhancement in the arterial phase, and wash out in the venous phase. The mass was anterior to the aorta and inferior vena cava (IVC) and was anteromedial to the right kidney, abutting the fundus of the gall bladder, and reaching the level of porta hepatis (Figure 1C-F). There was evidence of parenchymal infiltration into the liver, but enlarged lymph nodes were not detected by CT. Based on these findings, a differential diagnosis of sarcoma was made.

Intraoperatively, a large, firm tumor, originating from the head of pancreas, was found pushing against the caudate hepatic lobe and IVC. Multiple reactive lymph nodes were also identified in frozen sections. An open biopsy showed that the mass was a neuroendocrine tumor. The tumor was resected using a pancreaticodudenectomy (Whipple procedure) and it included segment VI of the liver and a small segment of the IVC. During the operation, the patient’s blood pressure spiked (>220 mm Hg) when the tumor was manipulated. The increase in blood pressure was not transient and it could only be controlled through a continuous infusion of a combination of alpha and beta blockers, in addition to nitroglycerine, until the tumor was resected.

**Figure 2:**
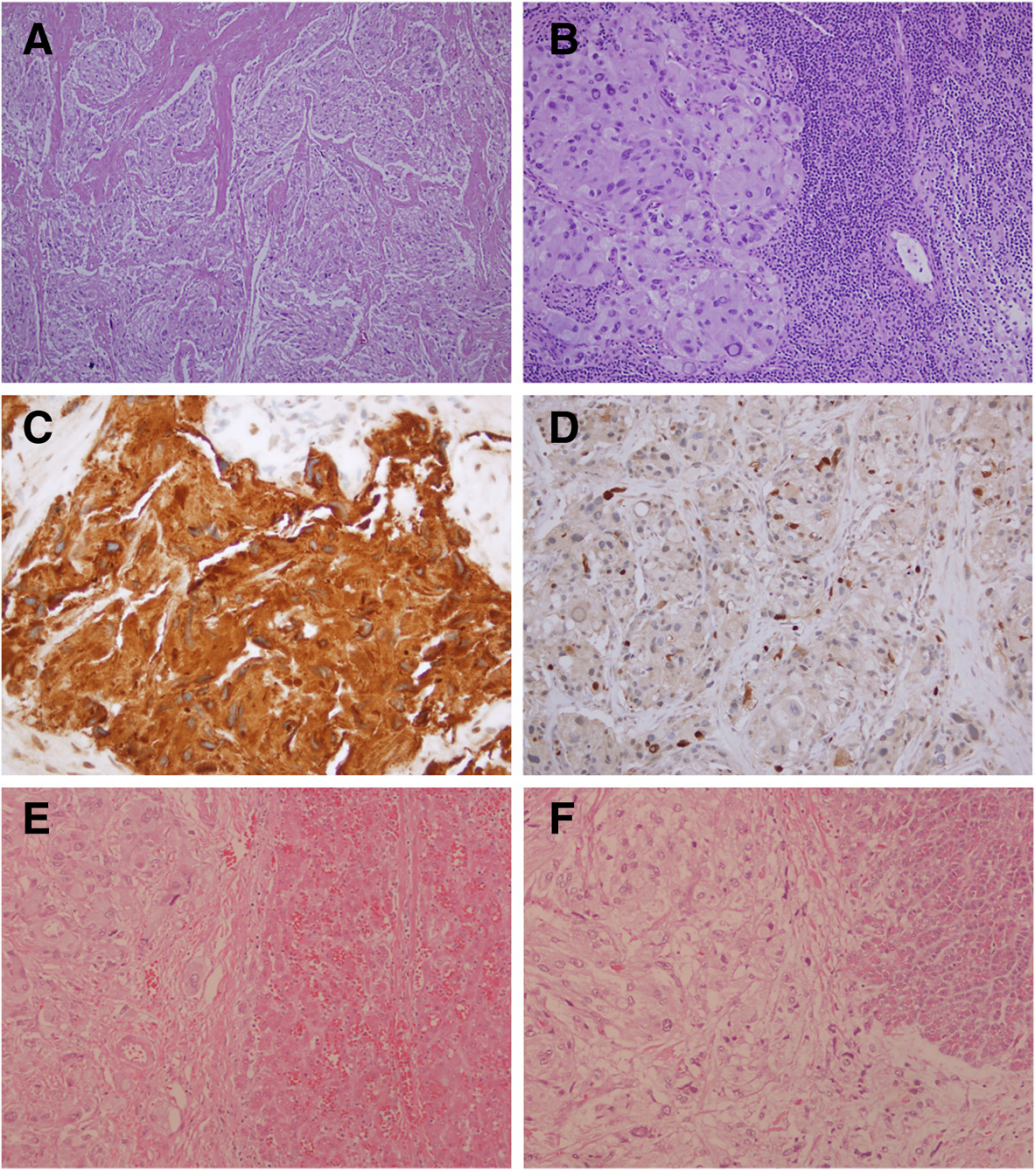
**Pathology slides of the resected specimen with different stains and sites. (A)** Hematoxylin and eosin stained section of the pancreas demonstrating the classical features of paragangiloma. **(B)** Lymph node demonstrating metastasis. **(C)** Diffuse cytoplasmic positivity for chromogranin. **(D)** S-100 immunohistochemical stain highlighting the sustenticular cells. **(E)** Hematoxylin and eosin stained section of the liver showing that the tumor is pushing onto the boundaries of the liver. **(F)** Hematoxylin and eosin stained section of the pancreas showing an infiltration into the pancreatic tissue.

The final pathology report described a 9-cm tumor, composed of well-defined nests of cuboidal cells separated by vascular fibrous septa, having a classic zellballen appearance, with lymphovascular invasion; immunohistochemistry was positive for synaptophysin and chromogranin. All the resection margins were negative, and only one of the 15 lymph nodes showing evidence of metastasis (Figure 2A-D). Histological staining showed that the tumor completely infiltrates the pancreas; however, it is only pushing into the boundaries of the liver tissue (Figure 2E-F).

Although the patient’s post-operative recovery was unremarkable, she was re-admitted with recurrent abdominal pain one month after being discharged. Further examination revealed an abdominal collection at the site of the resection. The site was drained under CT guidance and the patient received a therapeutic dose of I^131^-metaiodobenzylguanidine (MIBG). A subsequent total body scan revealed the absence of other associated neuroendocrine tumors and no evidence of multicentricity. Repeated follow-up MIBG scans were done, without evidence of recurrence. The patient has been disease free for >36 months.

## Conclusion

We report here a case of pancreatic paraganglioma, was identified through CT scan and histological evidences. We ruled out that the tumor was retroperitoneal because it was not attached to any structure and it fully mobilized with the head of the pancreas on kocherization, as a single mass. In addition, pathological findings showed that the tumor was very infiltrative and involved the entire pancreatic tissue. On the contrary, the tumor did not seem to originate from the liver because it covered only a small part of the liver as seen on the CT scan. It did not infiltrate into the liver tissue and was only pushing onto the boundary of the liver as seen on the pathology slide, which most likely means that it did not originate from the liver.

Pancreatic paragangliomas are extremely rare, with only 16 cases having been reported in the literature. Whether this tumor type is an extension of a retroperitoneal tumor of true visceral origin, derived from ectopic paraganglia, remains unknown [1,2]. The mean age of the 16 patients with pancreatic paragangliomas, reported in the literature, was 67 years (range, 42–85 years). Of those patients, 11 were women [1]. The patient in the present case was a woman and the youngest individual to have been diagnosed with pancreatic paraganglioma. Preoperative diagnosis of pancreatic paraganglioma is difficult unless the tumor is functional. However, pancreatic paragangliomas are generally non-functional as evidenced by the fact that only 1 of the previous 16 cases showed hormonal secretion [5]. Our patient presented with a painful upper abdominal mass but she was not hypertensive. However, her blood pressure spiked during the operation when the tumor was manipulated and was only controlled through a continuous infusion of alpha and beta blockers and nitroglycerine, raising the possibility of a functional tumor. Unfortunately, since we did not suspect the possibility of a functional tumor until we observed the marked increase in blood pressure at the time of resection and tumor manipulation, we did not measure the hormonal levels and we cannot be certain of the functional nature of the tumor.

The CT findings of pancreatic paragangliomas are different from those of pancreatic carcinomas. The latter appear as poorly enhanced masses with dilation of the bile and pancreatic ducts, as well as retroperitoneal involvement. In contrast, pancreatic paragangliomas, even when located at the head of pancreas, are not usually associated with biliary duct dilation and the patients are not usually jaundiced, which aids in differentiating pancreatic paraganglioma from pancreatic carcinoma [6,7]. In the present case, the patient was not jaundiced and her abdomino-pelvic CT scan showed a well-defined, hypervascular pancreatic mass with evidence of hepatic parenchymal infiltration, but a normal biliary tree. Hypervascularity is another common feature of paragangliomas of the pancreas and other sites [6].

Pancreatic paragangliomas have been reported in multiple locations throughout the pancreas, including the head (about two thirds of the reported cases), body, and tail [1,2,8]. In our study, the tumor originated from the head of pancreas and was large and firm. The present tumor, measuring 9 cm, is the largest known to have been reported, with typical tumor sizes ranging from 2–6 cm [1,4,8,9].

The pathology of these tumors is based on careful gross examinations of the specimens and histopathological studies based on hematoxylin-eosin sections. In addition, immunohistochemistry usually reveals these tumors to be positive for synaptophysin and chromogranin [10,11], as in the present case. Pheochromocytomas and extra-adrenal paragangliomas have unpredictable behavior and often metastasize late. Therefore, these tumors should never be considered malignant unless there is documented metastasis to the lymph nodes or other distant sites. However, extensive local invasion should be documented as it is commonly correlated with the metastatic potential of the tumor [11]. Mitotic activity, vascular invasion, and necrosis are histological features that are suggestive, but not diagnostic, of malignant behavior [10]. The pathologist has to retain a high index of suspicion to avoid interpreting separate primary tumors as lymph nodes [11].

Kimura et al. introduced a scoring system to predict the prognosis of pheochromocytomas and other extra-adrenal sympathetic paragangliomas. This scoring system depends on the tumor characteristics, including the tumor pattern (large, irregular nests, pseudo-rosettes), increased cellularity vascular invasion, coagulation necrosis, high proliferation index, and the type of catecholamine secretion [12]. The scores were stratified into well, moderate, and poorly differentiated categories and the risk of metastasis was found to increase when the tumor became less differentiated. In the present case, the final pathology report described a tumor with lymphovascular invasion. Immunohistochemistry revealed that the tumor was positive for the presence of synaptophysin and chromogranin with one metastatic lymph node. This positive lymph node and the lymphovascular invasion provided clear evidence of the malignant nature of the tumor, making the present case the second malignant case reported in the literature. The first was reported by Higa and Kapur where there was one positive lymph node out of 44 studied specimens [1]. None of the other cases have demonstrated lymph node or distant metastases. Similarly, none of the other case follow-ups described the development of metastases in high risk patients.

Although most paragangliomas are solitary and typically arise sporadically, they can be multicentric or hereditary. Therefore, a screen for additional tumors is necessary since they may be considered as part of multiple endocrine neoplasms [1,2,13]. In addition, the possibility of malignant transformation of paragangliomas makes surgical excision the treatment of choice, and aggressive surgery is mandatory to obtain disease-free survival [13]. For pancreatic lesions, pancreaticoduodenectomy is recommended for tumors of the pancreatic head; for other locations, distal or even total pancreatectomy may be indicated, according to the tumor extension [13,14]. In this case, a pancreaticoduodenectomy was performed and the patient had an uneventful postoperative course. Postoperative radiation therapy has also been advocated in cases proven to be malignant [15]. Therapy with radionucleotides may be used for tumors exhibiting uptake on diagnostic scans, with I^131^-MIBG radiotherapy being the treatment of choice. Chemotherapy is reserved for more advanced disease stages. Even in advanced cases, recent genetic studies have suggested that the use of targeted therapy may be useful, but additional validation is still required [15]. In the present case, the patient received a therapeutic dose of I^131^-MIBG one month after the operation, and remains disease-free after more than 36 months.

In conclusion, pancreatic paragangliomas are rare tumors, with only 16 cases reported to date. The patient in the present case was the youngest among the reported cases and the tumor was the largest reported. In addition, this is the second case with lymph node metastasis ever to be reported.

## Consent

Written informed consent was obtained from the patient for publication of this Case report and any accompanying images. A copy of the written consent is available for review by the Editor of this journal.

## Abbreviations

CT: Computed tomography; IVC: Inferior vena cava; MIBG: Metaiodobenzylguanidine.

## Competing interests

The authors declared that they have no competing interests.

## Authors’ contributions

BA: The treating physician who supervised the entire case and wrote the paper. YA: The resident responsible for following up with the patient and collecting the data and images. In addition, he contributed substantially in reviewing published cases and writing the paper. SK: Assisted in the surgery and followed up the patient pre- and post-operatively. She also contributed in writing the paper and doing literature review. MH: The pathologist who worked on the slides and generated the diagnosis. MH: Wrote the paper. All authors read and approved the final manuscript.

## Pre-publication history

The pre-publication history for this paper can be accessed here:

http://www.biomedcentral.com/1471-2407/13/486/prepub
